# Pentiptycene-Based Luminescent Cu (II) MOF Exhibiting Selective Gas Adsorption and Unprecedentedly High-Sensitivity Detection of Nitroaromatic Compounds (NACs)

**DOI:** 10.1038/srep20672

**Published:** 2016-02-09

**Authors:** Minghui Zhang, Liangliang Zhang, Zhenyu Xiao, Qinhui Zhang, Rongming Wang, Fangna Dai, Daofeng Sun

**Affiliations:** 1 State Key Laboratory of Heavy Oil Processing, China University of Petroleum (East China), College of Science, China University of Petroleum (East China), Qingdao Shandong 266580, People’s Republic of China

## Abstract

The assembly of a fluorescent pentiptycene-based ligand with copper ion resulted in the formation of a 3D porous metal-organic framework (**UPC-21**) based on well-known paddlewheel SBUs. **UPC-21** exhibits selective adsorption of CO_2_ over CH_4_ and N_2_ at 273 K and 295 K, C_2_H_2_ over CH_4_ at 273 K. The most significant performance of **UPC-21** is its highly efficient detection of NACs such as 4-NP, 1,4-DNB, NB, and 1,3-DNB with the calculated quenching constants, *K*_sv_, being 3.097 × 10^6^, 1.406 × 10^6^, 4.420 × 10^5^, and 1.498 × 10^5 ^M^−1^ for 4-NP, 1,4-DNB, NB, 1,3-DNB, respectively, which keeps a record on the fluorescence detection of NACs. This is the first porous Cu(II) MOF that exhibits fluorescent detection of NACs with high sensitivities.

The development of metal-organic frameworks (MOFs) has provided an excellent platform for rational design and synthesis of functional materials with desired properties^1^,^2^,^3^,^4^,^5^. Through careful design or select of organic ligand, functional MOFs with gas storage/separation, catalysis, and luminescent sensors were successfully constructed and reported in the past decades^6^,^7^,^8^,^9^,^10^,^11^,^12^,^13^. In particular, construction of MOF-based luminescent sensor has been received much attention of chemists due to its potential application in detection of nitroaromatic compounds (NACs). Nitroaromatics (NACs), which are composed of a benzene ring functionalized with several nitro-groups, have become serious pollution sources due to their explosivity and high toxicity. Beside, the structural tailorability and chemical versatility provide a significant level of tenability to detection of NACs with MOFs^14^. In general, it is highly dependent on the organic ligand or metal ion/cluster for the fluorescent behaviour of a MOF material. Hence, a large number of luminescent MOFs based on organic ligands with chromophores or metal ions such as Cd^2+^, Zn^2+^, Ln^3+^, or the combination of these two parts have been widely synthesized, and their application in fluorescent detection of NACs has been studied in recent years^15^,^16^,^17^,^18^,^19^,^20^,^21^. Surprisingly, study of fluorescent Cu(II) MOF on the application of sensing NACs remains unexplored, although porous Cu(II) MOFs with high gas uptake can be easily constructed by the assembly of carboxylate ligands with Cu(II) ions^22^,^23^,^24^,^25^. Furthermore, the detection sensitivity is somewhat low for the reported luminescent MOFs on the detection of NACs. Thus, construction of luminescent MOFs with high-sensitivity detection of NACs is still a great challenge to chemists.

On the other hand, organic ligands used for the construction of functional MOFs are limited in phenyl- or anthracene-based carboxylate or N-donor ligands^26^,^27^,^28^,^29^,^30^,^31^. However, the application of iptycene–based organic ligands in the assembly of porous MOFs is quite rare^32^. Actually, iptycene-based conjugated polymers were widely synthesized and studied, which exhibits excellent fluorescence sensing applications^33^. Moreover, the rigid iptycene groups can significantly prevent aggregation of the polymers to generate excellent properties^34^. Similar results should be achieved if the iptycene-based ligands are used to construct functional MOFs. Very recently, MOFs constructed from triptycene- and pentiptycene-based carboxylate ligands were reported by MacLachlan and coworkers^35^. However, porous MOFs based on pentiptycene-based ligands with gas uptake and fluorescent sensors remain unexplored to date.

Continuing our previous work by use of rigid tetracarboxylate ligands (Fig. 1a,b) to construct porous MOFs^36^,^37^,^38^,^39^,^40^, we further extended the central anthracene core to pentiptycene core to synthesize a new pentiptycene-based tetracarboxylate ligand, H_4_**L** (Fig. 1c). The solvothermal reaction between H_4_**L** and Cu(NO_3_)_2_∙3H_2_O resulted in the formation of a large amount of green crystals. Single-crystal X-ray diffraction, elemental analysis and TGA measurements reveal that the formula of the complex is [Cu_3_(**L**)_1.5_(H_2_O)_3_]∙3DEF∙20H_2_O (**UPC-21**). **UPC-21** exhibits selective adsorption of CO_2_ and highly efficient detection of NACs. To the best of our knowledge, the sensitivity of **UPC-21** on the detection of NACs keeps a record among the reported MOF-based fluorescent sensors.

## Results and Discussion

### Crystal structure of UPC-21

**Figure 2:**
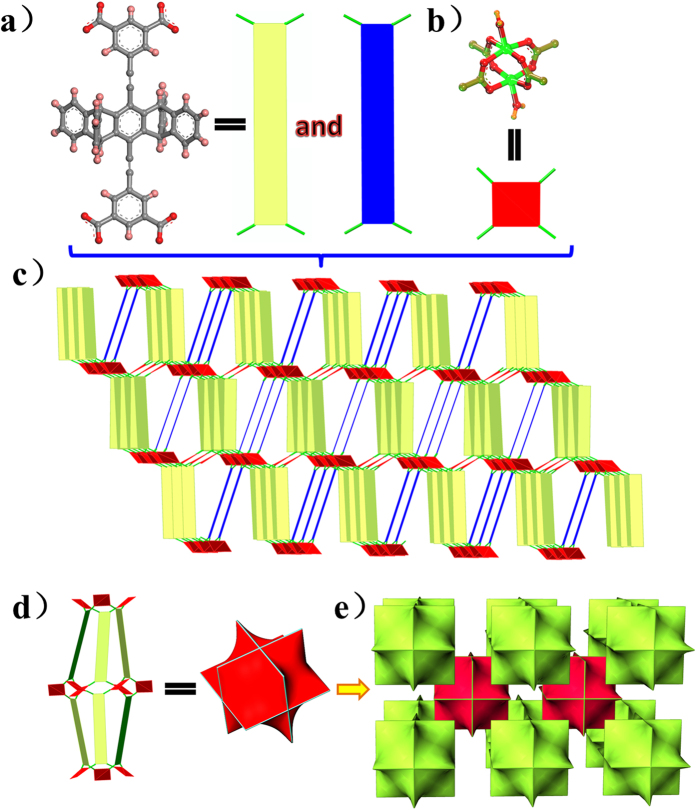
Crystal structure of UPC-21. **(a)** the square-planar linker of **L**^4−^ ligand in **UPC-21**. **(b)** The square-planar node of the paddlewheel SBU. **(c)** The NbO topological net. **(d)** Schematic representation of the linkage among the spindle-shaped cages.

Single crystal X-ray diffraction analyses reveal that **UPC-21** crystallizes in the monoclinic *C*2/*c* space group and is a 3D porous framework based on Cu_2_(COO)_4_ paddlewheel SBUs. There are three Cu^2+^ ions, three halves of **L**^4−^ ligands, and three coordinated water molecules in the asymmetric unit. Hence, the paddlewheel SBUs are connected by the backbone of **L**^4−^ ligands to generate a 3D porous framework. If the tetracarboxylate ligand of **L**^4−^ can be considered as a 4-connected planar linker and the paddlewheel SBU as a square planar node, the present framework is a NbO network with the short Schläfli vertex notation of the net being {6^4. 8^2}. Indeed, the framework contains spindle-shaped cages and each cage is connected with other eight similar cages. Thus, **UPC-21** can also be considered as formed by the infinite connection of the spindle-shaped cages (Fig. 2). The total accessible volume in **UPC-21** is 54.2% using the PLATON/VOID routine^41^, after the removal of the axial coordinated water molecules of the paddlewheel SBU.

### Gas sorption properties

**Figure 3:**
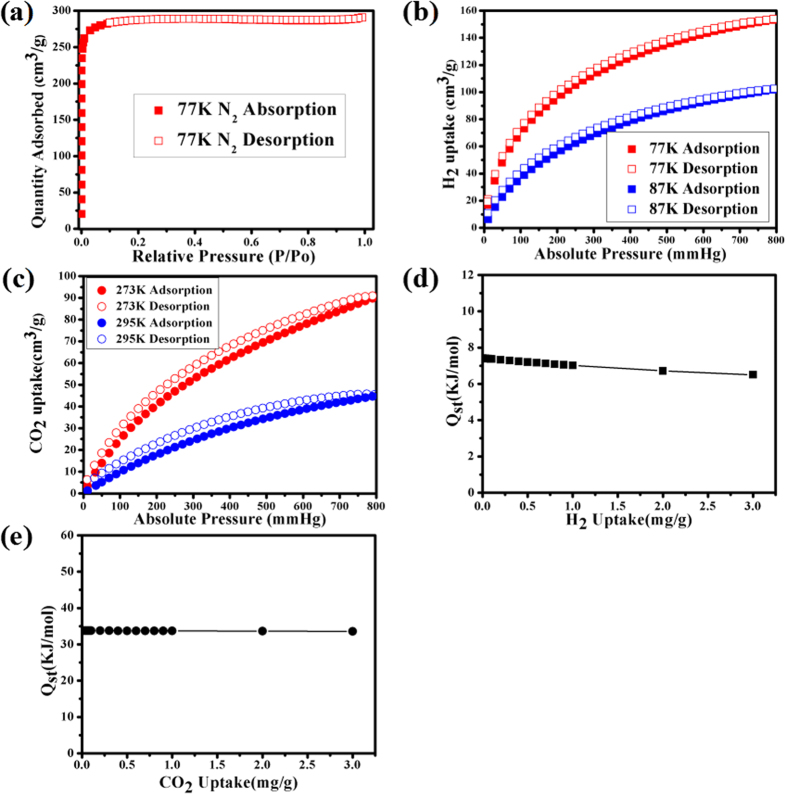
Gas uptakes for UPC-21. **(a)** The N_2_ sorption isotherms for **UPC-21** at 77 K. **(b)** The H_2_ adsorption capacity for **UPC-21** at 77 K and 87 K. **(c)** The CO_2_ adsorption capacity for **UPC-21** at 273 K and 295 K. **(d)** The *Q*_st_ of **UPC-21** for H_2_. **(e)** The *Q*_st_ of **UPC-21** for CO_2_

In order to confirm the permanent porosities of **UPC-21**, various gas adsorption isotherms of **UPC-21** were measured under various temperature. Before the measurement, the freshly prepared sample of **UPC-21** was extracted by soxhlete extraction with acetone for 36 hours, then activated at 80 °C to generate solvent-free sample of **UPC-21**. As shown in Fig. 3, desolvated **UPC-21** displays a typical Type-I adsorption isotherm with the Brunauer-Emmett-Teller (BET) surface area and Langmuir surface area being 1117.0 and 1253.6 m^2^g^−1^, respectively. Low-pressure H_2_ and CO_2_ uptakes of desolvated sample of **UPC-21** were also determined using volumetric gas adsorption measurements, which exhibit the classical reversible Type-I isotherms. The type of adsorption suggests typically microporous having been retained after the removal of guest molecules. The PXRD pattern is consistent with that of the pristine sample, which also indicates that the structure of **UPC-21** is relatively stable after removing the guest molecules. Under the conditions of 77 K and 1 bar, the desolvated **UPC-21** has a maximum H_2_ uptake of 154 cm^3^ g^−1^. The H_2_ isosteric heat of adsorption (*Q*_st_) for **UPC-21** was calculated by fitting the H_2_ adsorption isotherms at 77 K and 87 K to a Virial-type expression. At zero coverage, the *Q*_st_ has the estimated value of 7.4 kJ mol^−1^, which is comparable to other MOF-5 series^42^,^43^. CO_2_ measurement for the desolvated **UPC-21** indicates that the maximum CO_2_ uptake is 86.7 cm^3^ g^−1^ under 273 K and 1 bar, and the corresponding *Q*_st_ of 33.6 kJ mol^−1^ was calculated by fitting the CO_2_ adsorption isotherms at 273 K and 295 K to a Virial-type expression. The *Q*_st_ value is much higher than other reported MOF materials^44^, indicating that the framework of **UPC-21** possesses high affinity to CO_2_ molecules.

### Selective adsorption

**Figure 4:**
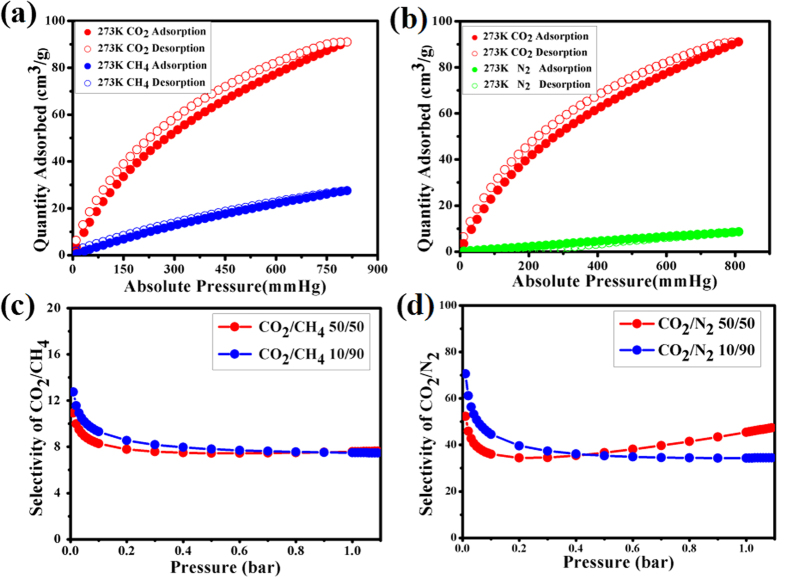
Selective gas adsorption for UPC-21. The CO_2_/CH_4_ (**a**) and CO_2_/N_2_ (**b**) sorption isotherms for **UPC-21** at 273 K. The CO_2_/CH_4_ (**c**) and CO_2_/N_2_ (**d**) selectivities for **UPC-21** at 273 K calculated by the IAST method for two CO_2_ concentration.

To further verify the selectivity potential to CO_2_ over N_2_ and CH_4_, the adsorption isotherms of CO_2_, CH_4_, and N_2_ were measured at 273 K (Fig. 4) and 295 K (Figure S5). As shown in Fig. 4, the maximum CO_2_, CH_4_, and N_2_ uptakes of 86.7, 25.4, and 7.8 cm^3^ g^−1^, respectively, were found at 273 K and 1 atm. To further evaluate the feasibility of the separations, ideal adsorbed solution theory (IAST) was performed on the basis of the experimentally recorded adsorption isotherms of **UPC-21**. According to the calculation results over the 10:90 and 50:50 CO_2_/CH_4_ or CO_2_/N_2_ mixed gas, the CO_2_/CH_4_ selectivities are 12.7–7.4 and 10.9–7.5, and the CO_2_/N_2_ selectivities are 70.5–34.3 and 52.2–47.4 for 10:90 and 50:50 mixtures, respectively. These results further indicate that **UPC-21** exhibits highly selective adsorption of CO_2_ over CH_4_ and N_2_ at 273 K^45^.

**Figure 5:**
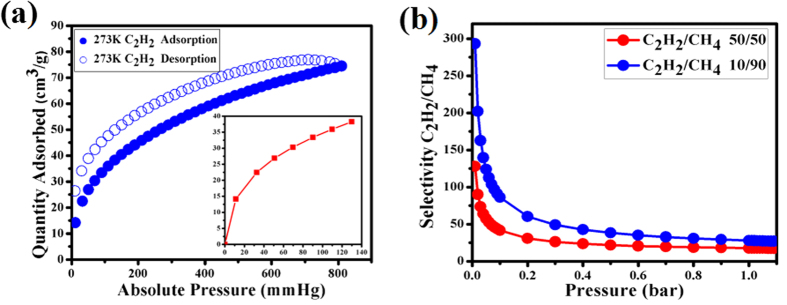
**(a)** The C_2_H_2_ sorption isotherms for UPC-21 at 273 K. **(b)** Selectivities for C_2_H_2_/CH_4_ at 273 K calculated by the IAST method.

The separation of CH_4_/C_2_H_2_ is also significant in the industrial process. Therefore, the gas separation task becomes increasingly challenge in recent years^46^,^47^,^48^. As shown in Fig. 5(a), the maximum C_2_H_2_ uptake is 72.6 cm^3^ g^−1^ at 273K and 1 atm, compared to CH_4_ uptake of 25.4 cm^3^ g^−1^. The selectivity for C_2_H_2_/CH_4_ mixture was calculated using the ideal adsorbed solution theory (IAST) as well. Figure 5(b) presents the IAST- derived selectivity of C_2_H_2_/CH_4_ for **UPC-21**. Notably, **UPC-21** exhibits substantially high selectivity at very low pressure. The C_2_H_2_/CH_4_ selectivities are 293.2–91.5 and 128.0–43.7 for 10:90 and 50:50 when the pressure is lower than 0.1 bar.

### Fluorescent property and NACs sensing

Fluorescent detection of NACs has received much attention of chemists. In the past decade, a large number of fluorescent MOFs were synthesized and reported. Although these fluorescent MOFs exhibit excellent sensing of NACs, the detection sensitivity needs to be further improved.

**Figure 6:**
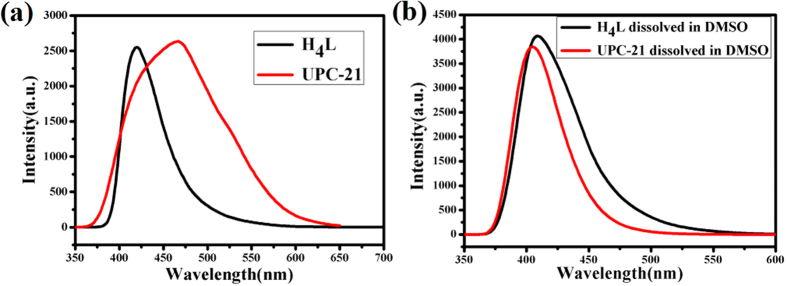
Emission spectra for H_4_L and UPC-21. **(a)** The solid state emission spectra for free ligand H_4_**L** and **UPC-21** at room temperature. **(b)** The emission spectra for H_4_**L** dissolved in DMSO and **UPC-21** dispersed in DMSO at room temperature.

Considering the excellent fluorescent property of pentiptycene-based ligand, the photoluminescence of **UPC-21** in the solid state was studied at room temperature. As shown in Fig. 6a, **UPC-21** exhibits a luminescent emission peak at 465 nm, upon excitation at 330 nm. The emission band of **UPC-21** can be ascribed to the organic linker, because similar emission at 418 nm was observed for the free H_4_**L** ligand. However, it is noteworthy that the change of luminescence for the **UPC-21**with free ligand under the same conditions, which probably can be considered as arising from strong coordination interactions between the ligand and metal^49^.

**Figure 7:**
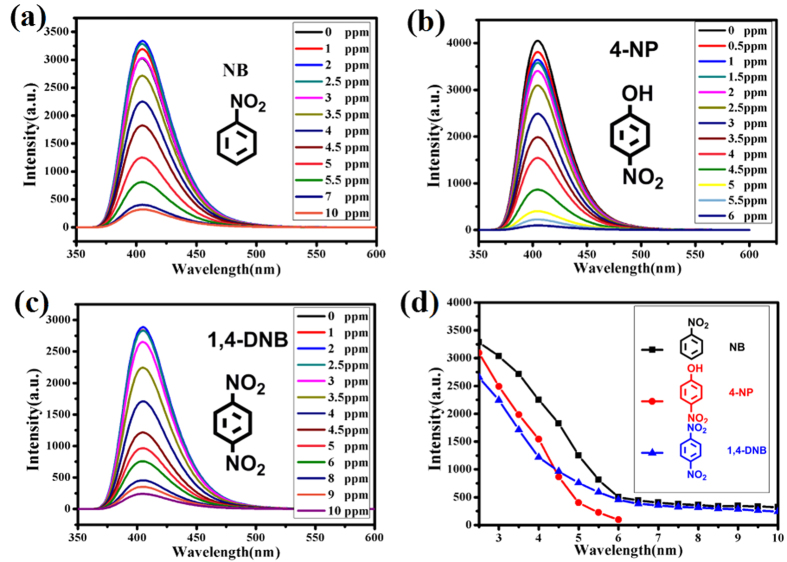
The emission spectra of **UPC-21** titrated with NB (**a**), 4-NP (**b**), 1,4-DNB (**c**), and the relationships between emission intensities and concentrations for NB, 4-NP, and 1,4-DNB (**d**).

To verify **UPC-21** sensing ability to NACs, fluorescent titrations were carried out with the gradual addition of analytes in DMSO to **UPC-21** dispersed in DMSO at room temperature. The simplest nitroaromatic compound, nitrobenzene (NB), was first chosen as the analyte. Hence, **UPC-21** dispersed in DMSO was titrated with NB in DMSO at room temperature, and the fluorescence change was monitored by PL spectroscopy. Interestingly, the fluorescent intensity increased slightly with the incremental addition of NB, but decreased significantly after further addition of NB at 3 ppm, indicating that the introduction of NB produced significant quenching of fluorescence intensity of **UPC-21**. The fluorescence intensity decreased to 89% at 10 ppm, and no further quenching was observed after 10 ppm (Fig. 7a), indicating that **UPC-21** can sense NB molecule with high sensitivity.

**Figure 8:**
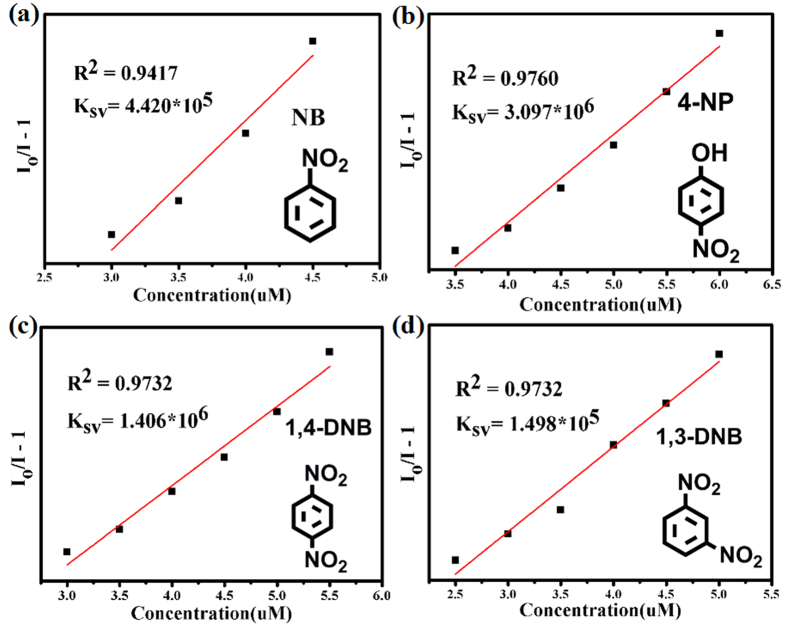
The Stern–Volmer plots for **UPC-21** with NB (**a)**, 4-NP (**b**), 1,4-DNB (**c**) and 1,3-DNB (**d**) in the relatively high concentration region. The solid lines represent fit to the concentration-resolved data using the Stern–Volmer equation.

Efficient sensing of NB prompted us to investigate the potential of **UPC-21** towards sensing other NACs such as 4-nitrophenol (4-NP), 1,4-dinitrobenzene (1,4-DNB), 1,3-dinitrobenzene (1,3-DNB), 4-Nitroaniline(4-NA), and p-nitrotoluene (4-NT). As expected, **UPC-21** exhibits efficient sensing of 4-NP with high sensitivity. When only 6 ppm of 4-NP was added to **UPC-21** dispersed in DMSO, nearly 98% of the initial fluorescence intensity was quenched. As contrast, the maximum fluorescence intensity of **UPC-21** was decreased by 91, 69, 16 and 15% upon the addition of 1,4-DNB, 1,3-DNB, 4-NT, and 4-NA at 10 ppm, respectively (Fig. 7 and Figure S6). These results indicate that **UPC-21** can efficiently sense NB, 4-NP, 1,4-DNB, and 1,3-DNB, in which 4-NP and 1,4-DNB can be detected most effectively. In order to further compare the efficiency of the sensor, the fluorescence quenching efficiency was calculated by use of the Stern-Volmer (SV) equation, *I*_0_/*I* = 1 + *K*_sv_[A], where *I*_0_ is the initial fluorescence intensity before the addition of analyte, *I* is the fluorescence intensity after adding the analyte as quencher, [A] is the molar concentration of analyte, and *K*_sv_ is the quenching constant (M^−1^). In particular, the quenching constant, *K*_sv_, can reflect the efficiency of the sensor. Based on the equation, if the *I*_0_/*I* vs [A] plot is linear, then *K*_sv_ can be estimated accurately At very low concentrations, the changes of the fluorescence intensity are very slight for 4-NP, NB, 1,3-DNB, and 1,4-DNB. The Stern-Volmer plots are nearly linear at relatively high concentrations for all analytes. The calculated quenching constants, *K*_sv_, are 3.097 × 10^6^, 1.406 × 10^6^, 4.420 × 10^5^, 1.498 × 10^5 ^M^−1^ for 4-NP, 1,4-DNB, NB, 1,3-DNB, respectively, which indicate that the quenching efficiency for the NACs in DMSO is the following order: 4-NP > 1,4-DNB > NB > 1,3-DNB (Fig. 8). The linear range for NACs detection is 2.5–6 ppm. Recently, several fluorescent MOFs with sensing of 4-NP, 1,4-DNB, NB, and 1,3-DNB were synthesized and reported. As shown in Table 1, **UPC-21** exhibits the highest sensitivity among the reported results. Furthermore, the detection limit (D) was calculated based on the equation: D = 3σ/k,(σ: standard, k:slope). The calculated detection limits, D, are about 0.0775, 0.0896, 0.0949, and 0.123 ppm for 1,4-DNB, 4-NP, NB, and 1,3-DNB, respectively, which further confirm the high sensitivity of **UPC-21** on the fluorescent detection of 4-NP, 1,4-DNB, NB, and 1,3-DNB.

To further explore if **UPC-21** can sense other highly explosive NACs with three substituted groups, the fluorescent titrations for 2,4-dinitrotoluene (2,4-DNT) and trinitrophenol (TNP) were carried out. Surprisingly, **UPC-21** exhibits highly efficient sensing of TNP but no sensing for 2,4-DNT was observed (Figures S15 and S13). The maximum fluorescence intensity of **UPC-21** was decreased by 64% upon the addition of 6 ppm TNP. The calculated *K*_sv_ is about 5.209 × 10^5 ^M^−1^ (Figure S16), which is much higher than other reported results^50^,^51^,^52^,^53^,^54^,^55^.

It is known that the fluorescent detection of NACs by MOFs materials should derive from the truth that the fluorescence quenching phenomenon can occur as a result of the electron transfer from the framework of MOFs to the electron-deficient NACs molecules. The high-sensitivity detection of 4-NP and 1,4-DNB by **UPC-21** may result from the formation of strong interactions (such as π–π stacking) between the analytes and the side benzene rings of **L**^4−^ ligands after the analytes diffuse into the channels of the framework. In contract, when 1,4-DNB was added gradually to H_4_**L** dissolved in DMSO, there is no obvious changes for the fluorescence intensity (Figure S18), confirming that the analytes entered into the channels of **UPC-21** to interact with the framework. Beside, It should be note that **UPC-21** can sense highly explosive for 4-NP, which is possible owing to the presence of the OH^–^ group. With the involving of highly acidic OH^−^group, strong interaction is occurred via electrostatic interactions, which lead to the quenching effect maintained over a long range.

## Conclusions

In conclusion, a new 3D porous Cu (II) MOF (UPC-21) based on a fluorescent pentiptycene-based ligand was synthesized and characterized. UPC-21 shows selective adsorption of CO_2_ over CH_4_ and N_2_ at 273 K and 295 K, C_2_H_2_ over CH_4_ at 273 K, which possesses potential application on gas separation. Significantly, due to the existence of fluorescent ligand of H_4_L in the compound, UPC-21 exhibits fluorescence detection of 4-NP, 1,4-DNB, NB, and 1,3-DNB with high sensitivities. Furthermore, the calculated quenching constants, *K*_sv_, are 3.097 × 10^6^, 1.406 × 10^6^, 4.420 × 10^5^, 1.498 × 10^5 ^M^−1^ for 4-NP, 1,4-DNB, NB, 1,3-DNB, respectively, which keeps a record on the sensitivity of fluorescence detection of NACs. To the best of our knowledge, this work represents the first fluorescent Cu (II) MOF that exhibits high-sensitivity detection of nitroaromatic compounds such as 4-NP, 1,4-DNB. Further study will focus on the synthesis of fluorescent MOFs with other transition metal such as Zn^2+^ and Cd^2+^ *etc*. and H_4_L ligand, as well as their application on the fluorescence detection of NACs.

## Method

### Materials and measurements

All the chemical reagents were obtained from commercial sources and used without further purification. The ligand H_4_**L** was synthesized in 60% yield by a Sonogashira coupling reaction between bis-6,13-(4-acetenyl)pentiptycene and Dimethyl 5-iodoisophthalate followed by hydrolysis with dilute HCl. (Supporting Information, SI). Thermo-gravimetric analysis (TGA) experiments were carried out on a Mettler Toledo TGA instrument with a heating rate 10 °C /min at the range of 25–800 °C under a N_2_ atmosphere. Elemental analyses (C, H, N) were performed on a CE instruments EA 1110 elemental analyzer. The powder XRD data were obtained on an X-Pert PRO MPD diffractometer with Cu-Kα radiation. Photoluminescence spectra were recorded with a Hitachi F-7000 Fluorescence Spectrophotometer. Gas-sorption isotherms were carried out on the surface area analyzer ASAP-2020.

### Synthesis of UPC-21

Crystal of **UPC-21** was synthesized by solvothermal reaction of Cu(NO_3_)_2_∙3H_2_O (5.0 mg, 0.021 mmol) and H_4_**L** (2.0 mg, 0.0025 mmol) in the mixed solvents of N,N-diethylformamide (DEF) and water. The reaction system was heated to 75 °C for 1000 min and cooled to room temperature slowly. Green block crystals of **UPC-21** were obtained and dried in air at ambient temperature (yield: 25%, based on Cu). Elemental analysis calc. (%) for **UPC-21**: C, 54.58; H, 5.63; N, 1.99. Found: C, 53.13; H, 5.30; N, 2.02.

### Single-crystal X-ray diffraction study

Single crystal structure analysis of **UPC-21** was performed on Agilent Xcalibur Eos Gemini diffractometer with Enhance (Cu) X-ray Source Cu-Kα (λ = 1.54178 Å). An empirical absorption correction was used via the multi-scan method. The structure were solved by direct methods and refined by full-matrix least-squares on *F2* using *SHELXL-97* 1. The structure was examined using the Addsym subroutine of PLATON^2^ to assure that no additional symmetry could be applied to the models. Crystal and refinement parameters are listed in Table S1. CCDC No. 1418675 for **UPC-21**. These data can be obtained free of charge (http://www.ccdc.cam.ac.uk/data_request/cif). Full experimental details and crystallographic analysis are given in the Supplementary Information.

## Additional Information

**How to cite this article**: Zhang, M. *et al.* Pentiptycene-Based Luminescent Cu(II) MOF Exhibiting Selective Gas Adsorption and Unprecedentedly High-Sensitivity Detection of Nitroaromatic Compounds (NACs). *Sci. Rep.* **6**, 20672; doi: 10.1038/srep20672 (2016).

## Supplementary Material

Supporting Information

## Figures and Tables

**Figure 1:**
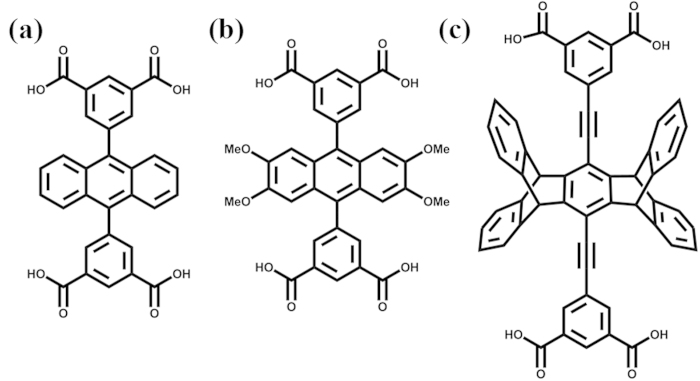
The tetracarboxylate ligands used in our previous works (**a**,**b**) and this work (**c**).

**Table 1:** Comparison of sensitivity of selected MOF-based sensors for the detection of NACs.

NACs	*K*_sv_/M^−1^	Reference
4-NP	2.2 × 10^4^	46
4-NP	7.22 × 10^4^	56
**4-NP**	**3.097 × 10**^**6**^	**UPC-21**
NB	2.2 × 10^2^	57
NB	1.89 × 10^3^	58
NB	2.843 × 10^3^	59
NB	4.13 × 10^3^	60
NB	9.5 × 10^2^	61
**NB**	**1.406 × 10**^**6**^	**UPC-21**
1,4-DNB	4.526 × 10^3^	59
**1,4-DNB**	**4.420 × 10**^**5**^	**UPC-21**
1,3-DNB	5.662 × 10^3^	59
1,3-DNB	1.5 × 10^3^	62
**1,3-DNB**	**1.498 × 10**^**5**^	**UPC-21**
