# Supplementary material for: Pentiptycene-Based Luminescent Cu (II) MOF Exhibiting Selective Gas Adsorption and Unprecedentedly High-Sensitivity Detection of Nitroaromatic Compounds (NACs)
Source: Sci Rep. 2016 Feb 9;6:20672. doi: 10.1038/srep20672 (PMC4746655; doi:10.1038/srep20672)
Supplement: Supporting Information [file srep20672-s1.doc]

**Supporting Information for**

# Pentiptycene-Based Luminescent Cu (Ⅱ) MOF Exhibiting Selective Gas Adsorption and Unprecedentedly High-Sensitivity Detection of Nitroaromatic Compounds (NACs)

Minghui Zhang,^‡^ Liangliang Zhang,^‡^ Zhenyu Xiao, Qinhui Zhang, Rongming Wang, Fangna Dai, Daofeng Sun*

State Key Laboratory of Heavy Oil Processing, China University of Petroleum (East China), College of Science, China University of Petroleum (East China), Qingdao, Shandong, 266580, People’s Republic of China.

Email: [dfsun@upc.edu.cn](mailto:dfsun@upc.edu.cn)

1. **Synthesis of ligand H_4_L.**

A mixture of bis-6,13-(4- acetenyl)pentiptycene **7** (prepared according to literature procedure.^S1^), Dimethyl 5-iodoisophthalate (1.6 g, 10 mmol), Trans-Dichlorobis(triphenyl- phosphine)Palladium(II) (0.7 g, 1.0 mmol), triphenylphosphine (1.0, 3.8mmol), CuI (0.08g, 0.42mmol) was loaded into a 250 mL Schlenk flask. About 150 mL Triethylamine were degassed and added through a canula. The solution was heated to 90 ℃ for 2 days, and then it was extracted with Trichloromethane. The mixture was washed with water and brine, then the organic layer was dried with MgSO_4_ and filtered, and the solution was dried on rotary evaporator. The residue was purified with chloroform through a short silica gel column (60% yield ).

The obtained ester was added in a mixture of THF (20 mL), MeOH (20 mL) and 2 M NaOH aqueous solution (5 mL) and stirred for 4 hours at room temperature. Then the solution was acidified with dilute HCl until the solution was at pH = 2. The resultant white precipitate was collected by filtration, washed with water, and dried to yield H_4_**L** (80%).





Scheme S1. Synthesis of ligand H_4_**L**.

^1^H NMR (500 MHz, DMSO, δ): 13.69 (s, 4H), 8.72 (d, 4H), 8.61 (s, 2H) 7.54 (q,8H), 7.00 (q, 4H) 6.12 (s, 4H)

^13^C NMR (101 MHz, DMSO, δ) 166.05, 144.46, 144.38, 136.34, 132.18, 130.32, 125.36, 124.01, 123.28, 113.79, 95.76, 85.02, 51.10.

ESI**-**TOF-HRMS: m/z calcd for C_54_H_30_O_8_: 806.19, found: 806.19


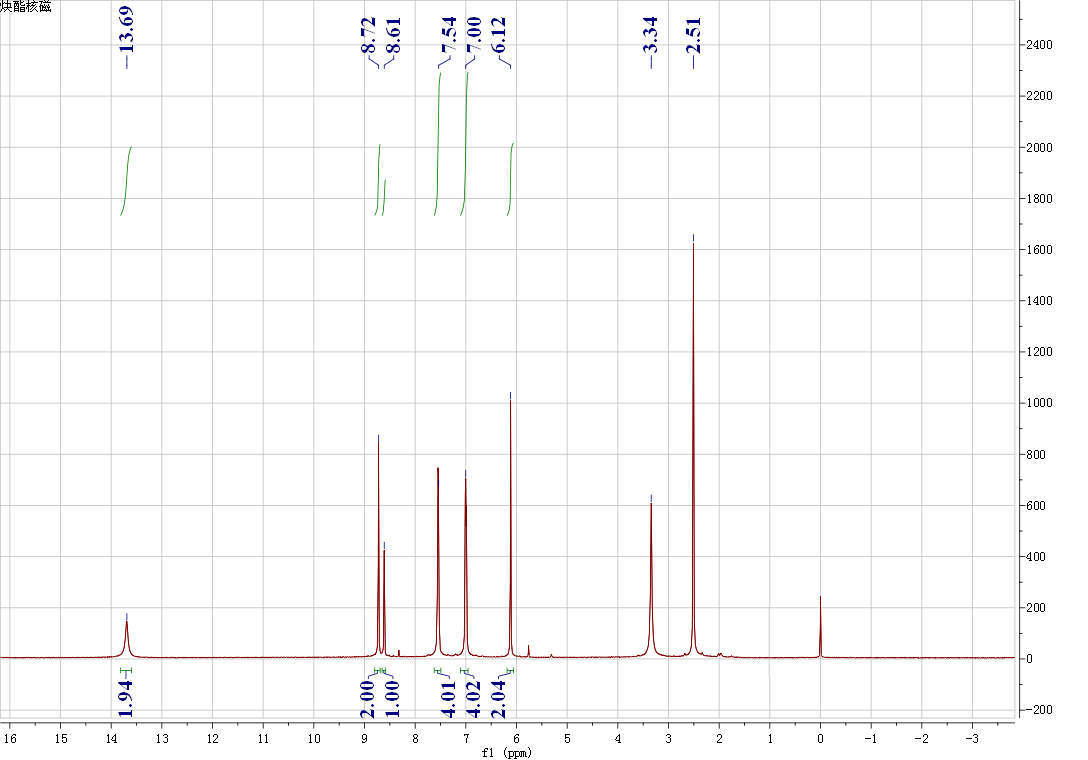


**Figure S1**. ^1^H NMR spectrum of H_4_**L**.


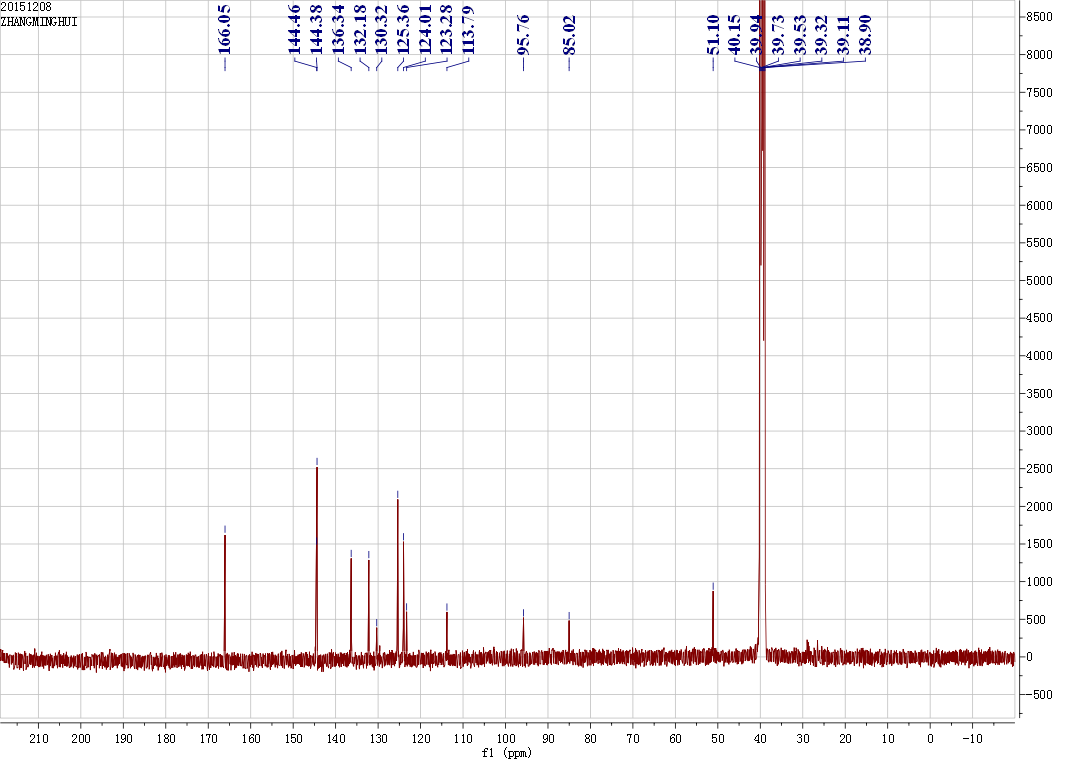


**Figure S2**. ^13^C NMR spectrum of H_4_**L**





**Figure S3**. ESI spectrum of H_4_**L**

1. **Powder X-ray diffraction a/nd thermogravimetric analysis**


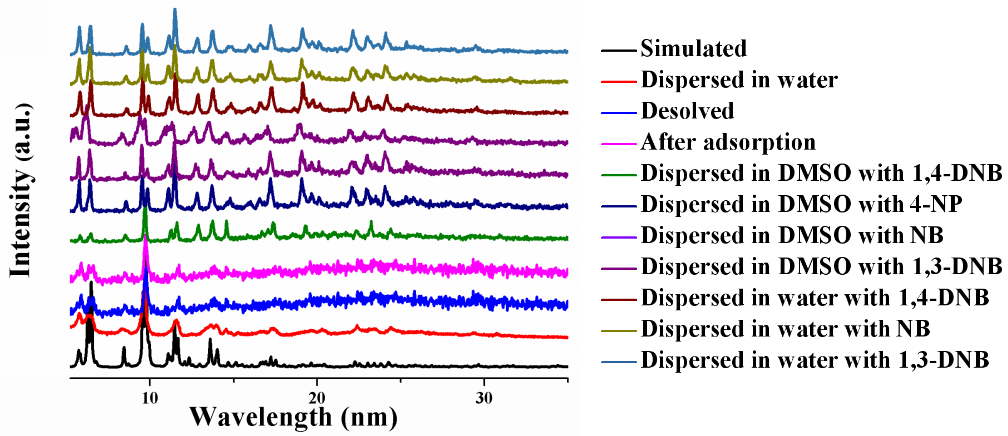


**Figure S4**. The PXRD patterns of **UPC-21**.

**

**

**Figure S5** TGA curves for **UPC-21**

1. **Single crystal X-ray diffraction analysis**

Single crystal structure analysis of **UPC-21** was solved by direct methods and refined by full-matrix least-squares on *F*^2^ using *SHELXL-97*.^S2^ The structure was examined using the Addsym subroutine of PLATON ^2^ to assure that no additional symmetry could be applied to the models. Crystal and refinement parameters are listed in Table S1.

Table S1: Crystal Data for UPC-21

| Identification code | **UPC-21** |
| --- | --- |
| Empirical formula | C_81_H_45_Cu_3_O_15_ |
| Formula weight | 1403.56 |
| Temperature/K | 293(2) |
| Crystal system | monoclinic |
| Space group | C2/c |
| a/Å | 32.0784(13) |
| b/Å | 18.4491(4) |
| c/Å | 41.2495(15) |
| α/° | 90.00 |
| β/° | 118.550(5) |
| γ/° | 90.00 |
| Volume/Å3 | 21443.8(12) |
| Z | 8 |
| ρcalcg/cm3 | 0.869 |
| μ/mm‑1 | 1.054 |
| F(000) | 5545.0 |
| Reflections collected | 35928 |
| Independent reflections | 18253 [Rint = 0.0391] |
| Data/restraints/parameters | 18253/0/895 |
| Goodness-of-fit on F2 | 1.385 |
| Final R indexes [I>=2σ (I)] | R1 = 0.1179, wR2 = 0.3385 |
| Final R indexes [all data] | R1 = 0.1318, wR2 = 0.3509 |
| Largest diff. peak/hole / e Å-3 | 1.15/-1.24 |

R_1_ = Σ| |F_o_|−|F_c_| |/ Σ|F_o_|, wR_2_ = [Σw(F_o_^2^−F_c_^2^)^2^]/ Σw(F_o_^2^)^2^]^1/2^

1. **Gas sorption**

The efficiency of **UPC-21** for CO_2_/CH_4_ CO_2_/N_2_ separation was calculated according to the IAST of Myers and Prausnitz along with the pure component isotherm, which determine the molar loadings in the mixture for specified partial pressures in the bulk gas phase. The absolute component loadings at 273 K were fitted with a single-site LangmuirFreundlich model (Equation 1).

(1)

Here, a is saturation capacity and b and c are constant. The fitting parameters of equation 1 as well as the correlation coefficients (R2) are listed in Table S2, S3.

Table S2. Equation parameters for the single-site Langmuir-Freundlich model.(CO_2_/CH_4_ )

| Adsorbates | a (mmol/g) | b (kPa^-1^) | c | R^2^ |
| --- | --- | --- | --- | --- |
| CO_2_ | 9.22678 | 0.01571 | 0.8322 | 0.99969 |
| CH_4_ | 4.74416 | 0.00385 | 0.96207 | 0.99994 |

Table S3. Equation parameters for the single-site Langmuir-Freundlich model (CO_2_/N_2_ ).

| Adsorbates | a (mmol/g) | b (kPa^-1^) | c | R^2^ |
| --- | --- | --- | --- | --- |
| CO_2_ | 9.22678 | 0.01571 | 0.8322 | 0.99969 |
| N_2_ | 2.20072 | 0.00154 | 1.05004 | 0.9999 |


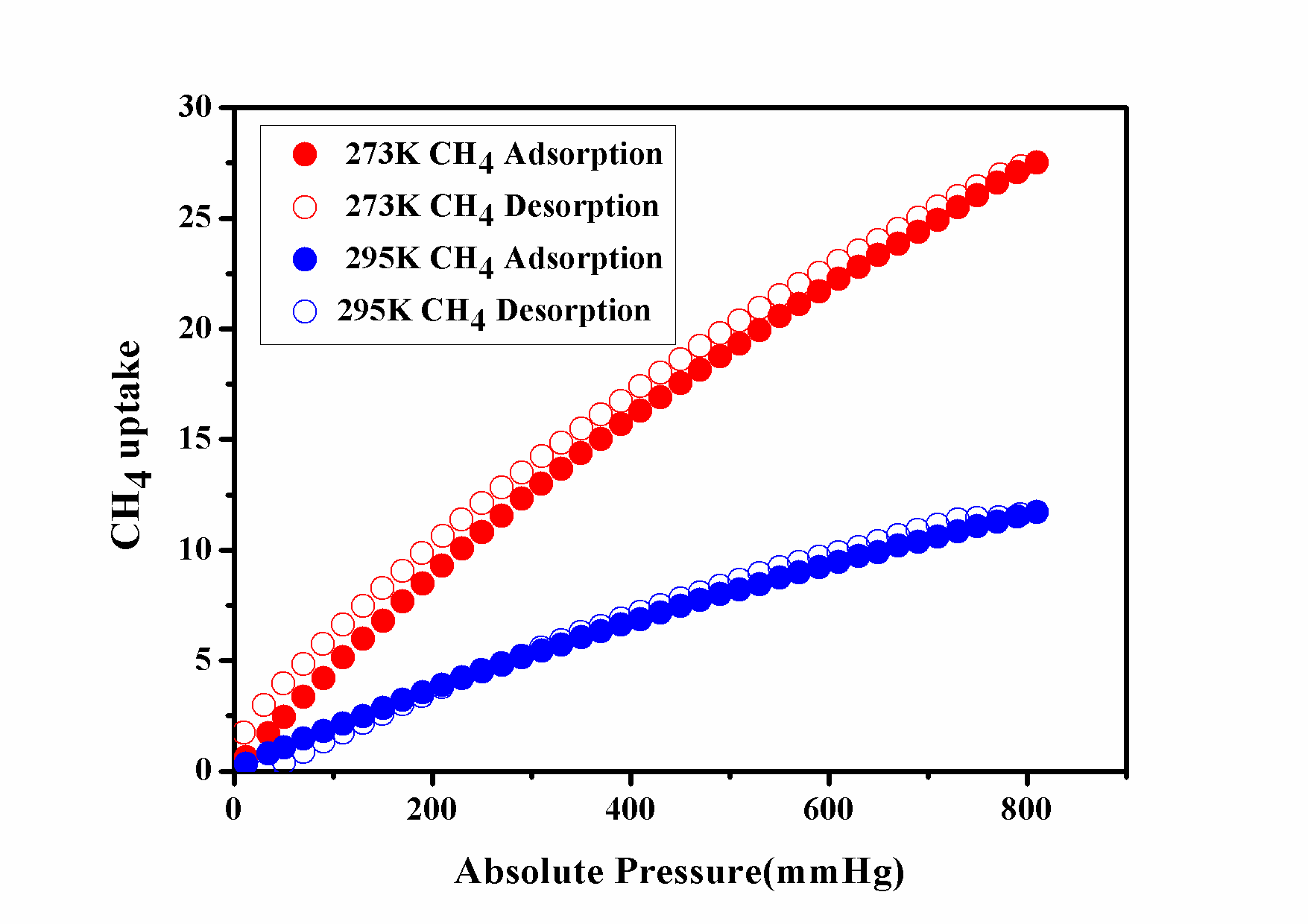


**Figure S6** The CH_4_ adsorption capacity for **UPC-21** at 273 K and 295 K.


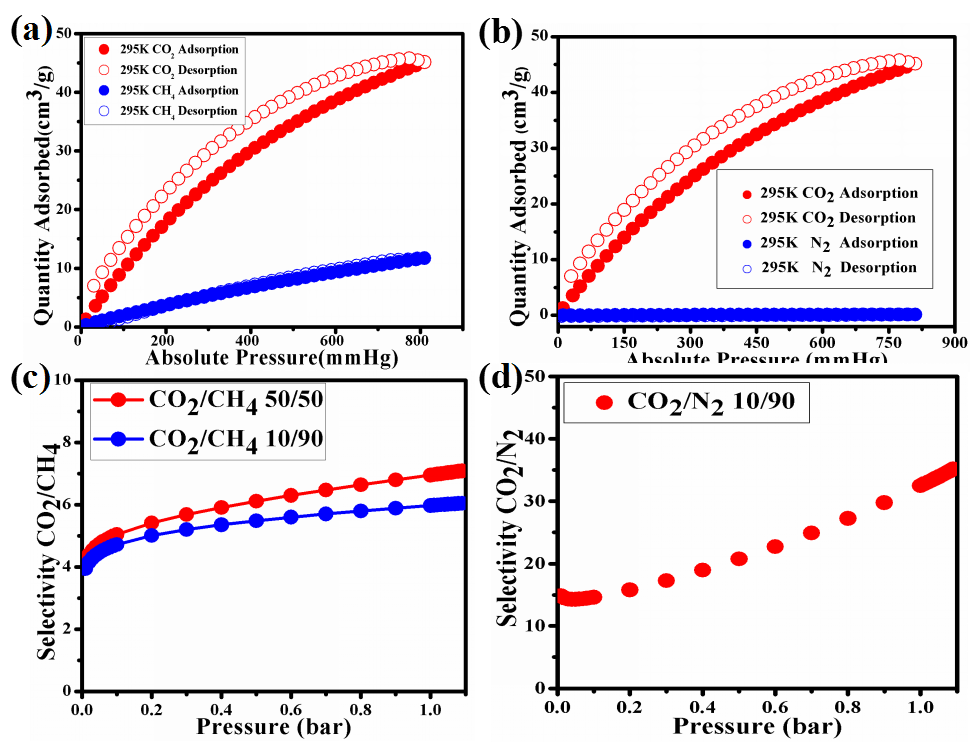


**Figure S7**. The CO_2_/CH_4_ (a) and CO_2_/N_2_ (b) sorption isotherms for **UPC-21** at 295 K. The CO_2_/CH_4_ (c) and CO_2_/N_2_ (d) selectivities for **UPC-21** at 295 K calculated by the IAST method for two CO_2_ concentration (CO_2_/CH_4_ 50/50 and 10/90) or CO_2_ concentration (CO_2_/N_2_: 10/90).

1. **Fluorescence study**


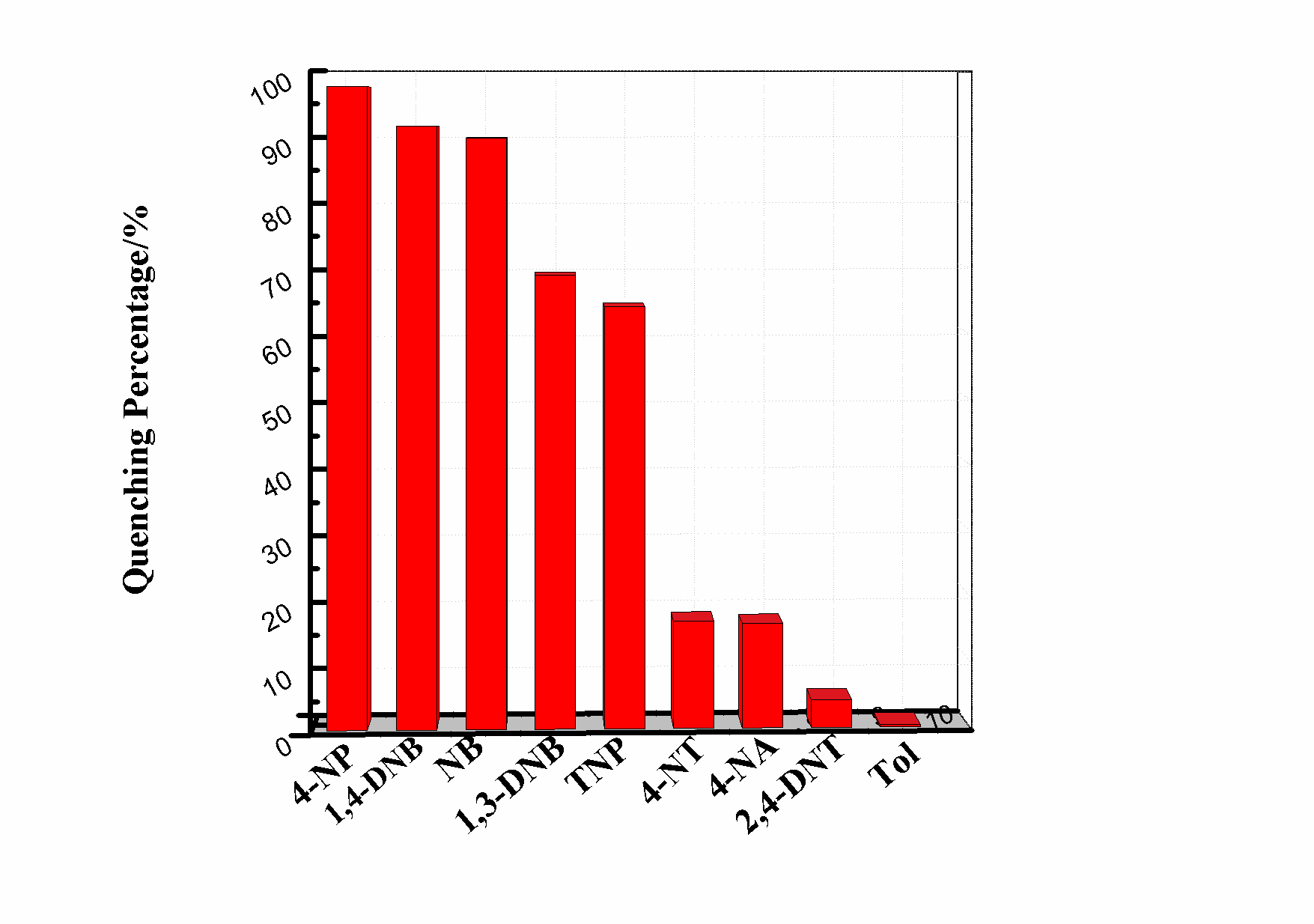


**Figure S8.** The percentage of fluorescence quenching upon the addition of the nitrobenzene derivatives .


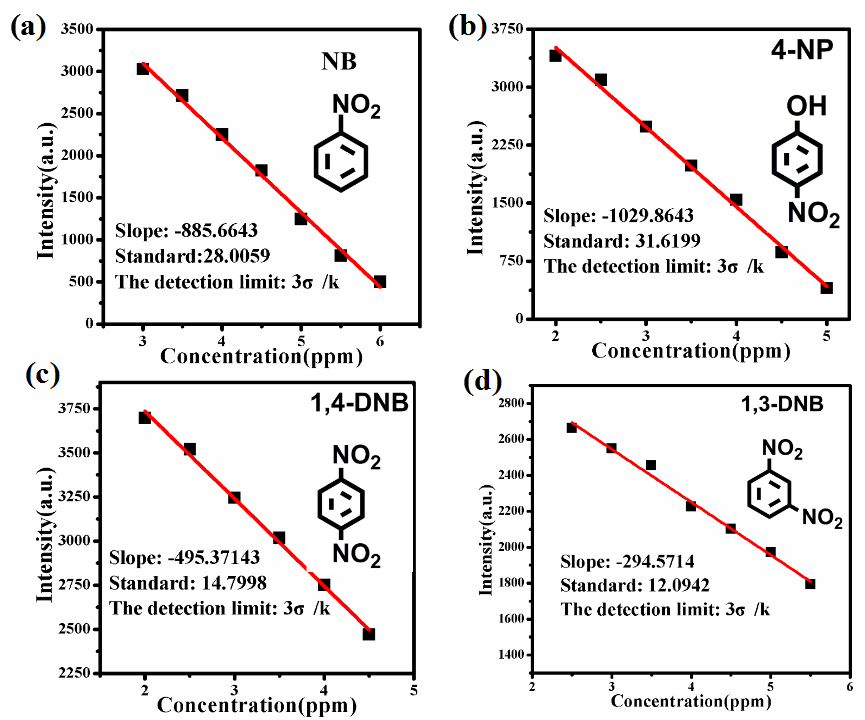


**Figure S9**. Detection limit (D) of NB(a), 4-NP(b), 1,4-DNB(c) and 1,3-DNB(d) for **UPC-21**.^S3^


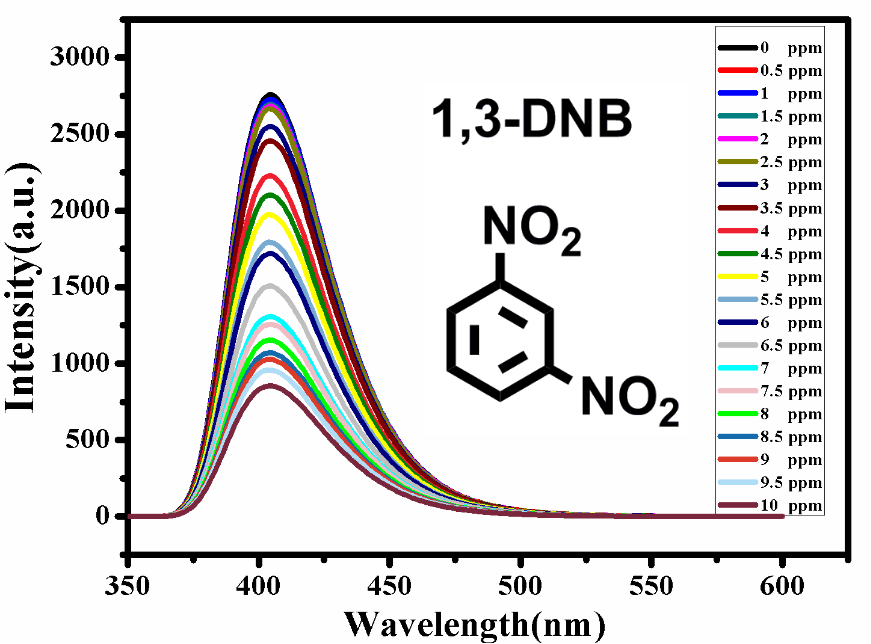


**Figure S10**. Effect on the emission spectra of **UPC-21** upon incremental addition of a 1,3-DNB solution (1mM) in DMSO.


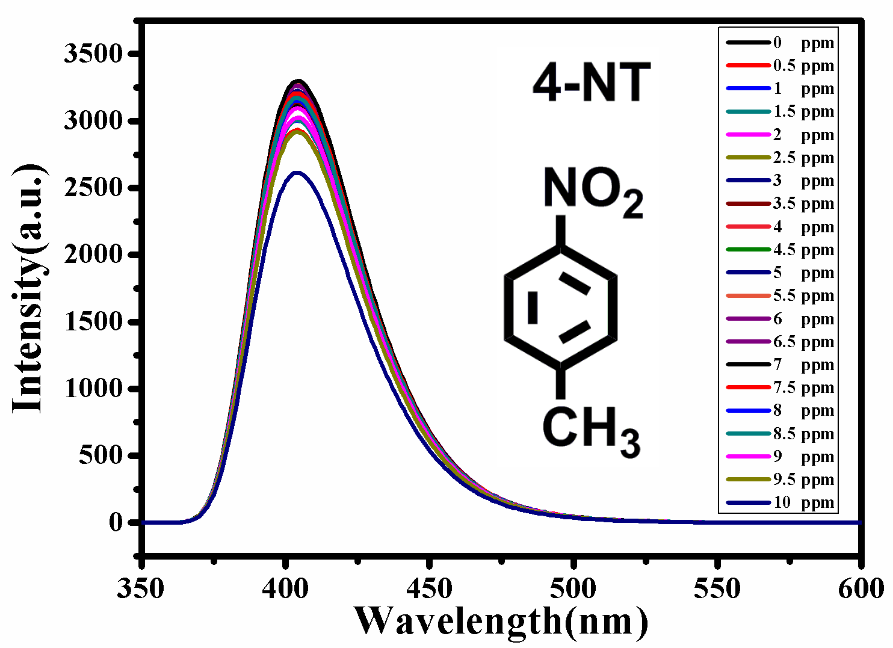


**Figure S11**. Effect on the emission spectra of **UPC-21** upon incremental addition of a 4-NT solution (1mM) in DMSO.


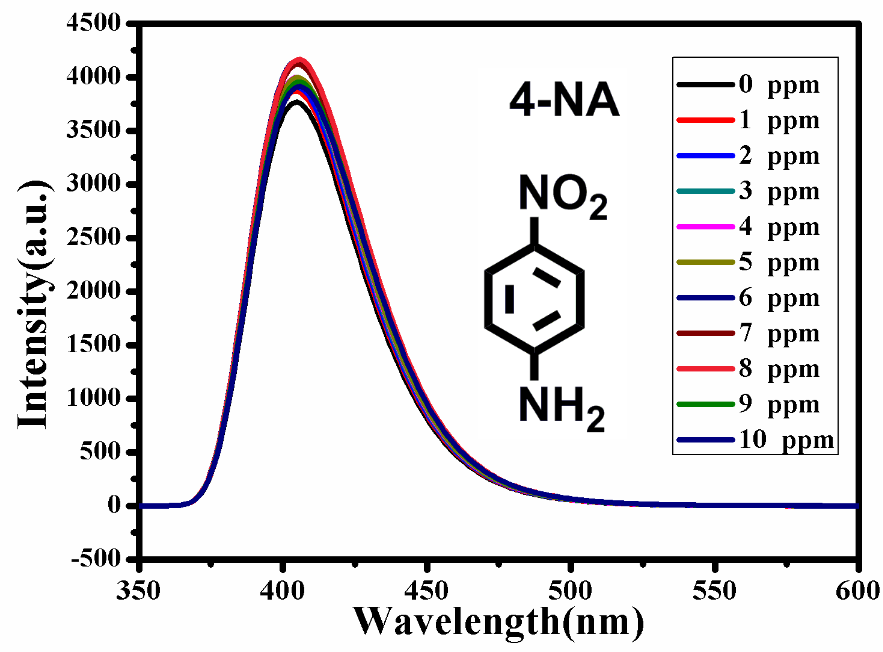


**Figure S12**. Effect on the emission spectra of **UPC-21** upon incremental addition of a 4-NA solution (1mM) in DMSO.


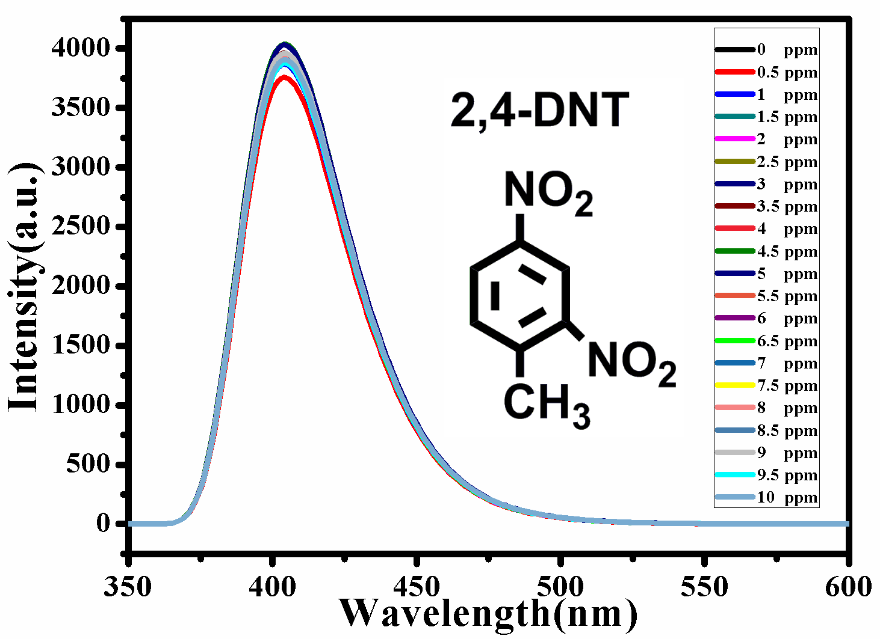


**Figure S13**. Effect on the emission spectra of **UPC-21** upon incremental addition of a 2,4-DNT solution (1mM) in DMSO.


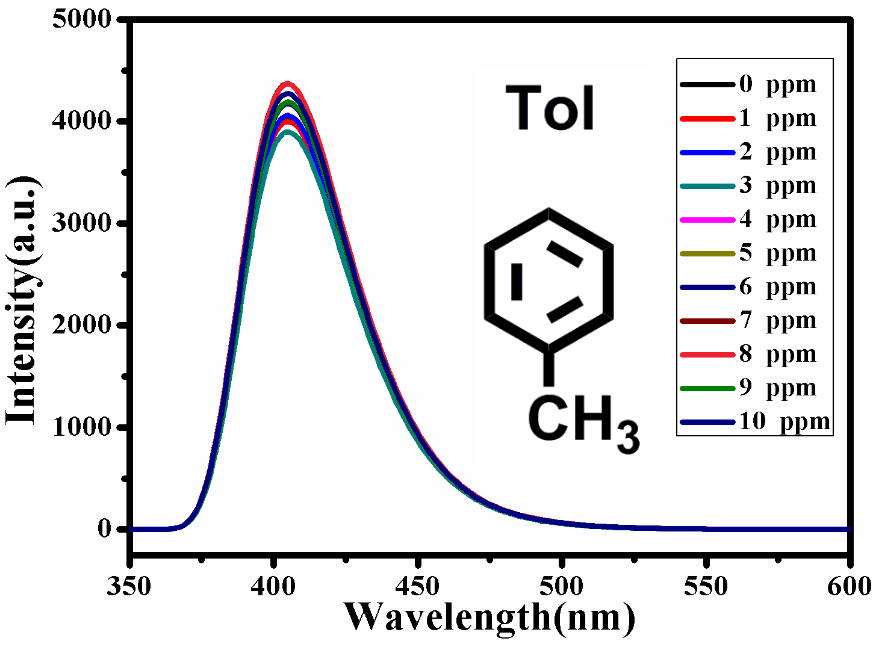


**Figure S14**. Effect on the emission spectra of **UPC-21** upon incremental addition of a Tol solution (1mM) in DMSO.


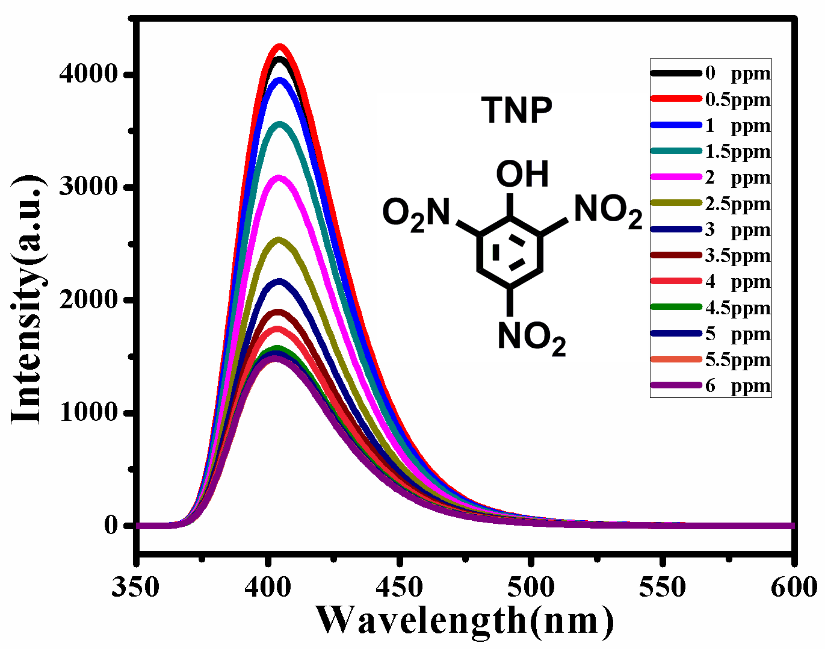


**Figure S15**. Effect on the emission spectra of **UPC-21** upon incremental addition of a TNP solution (1mM) in DMSO.


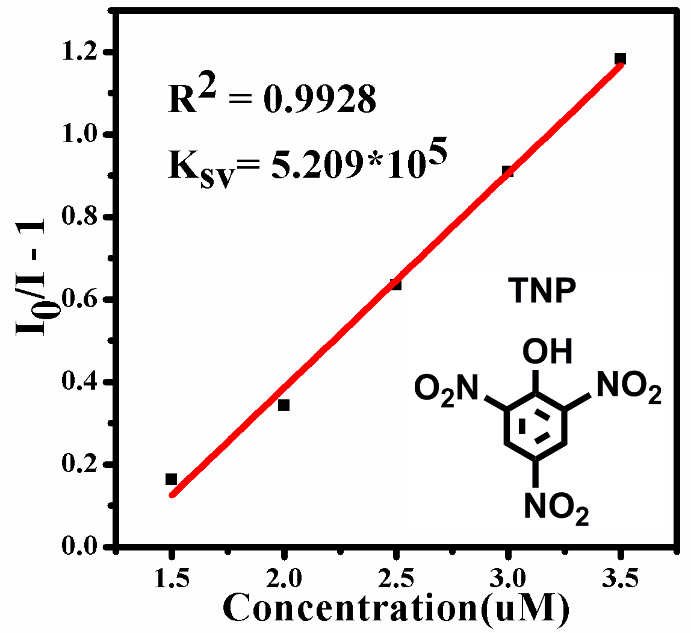


**Figure S16**. The Stern–Volmer plots for **UPC-21** with TNP.


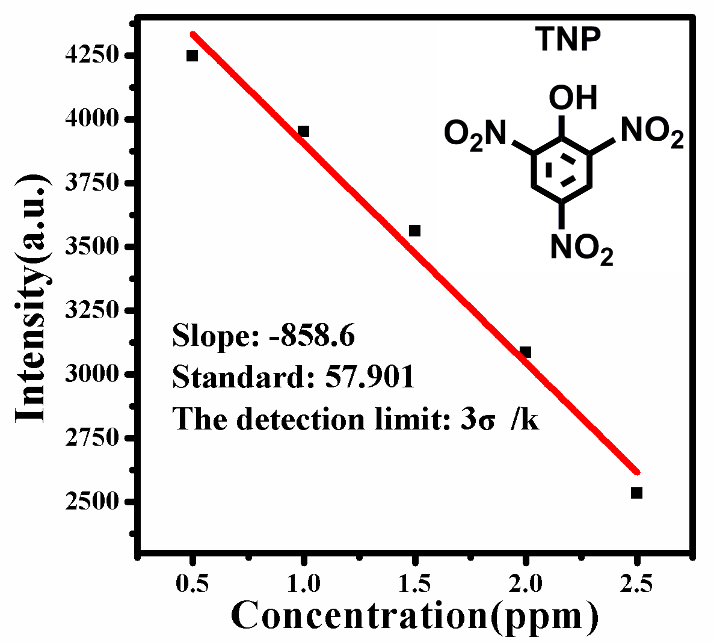


**Figure S17**. Detection limit (D) calculated was about 0.202 ppm for TNP.


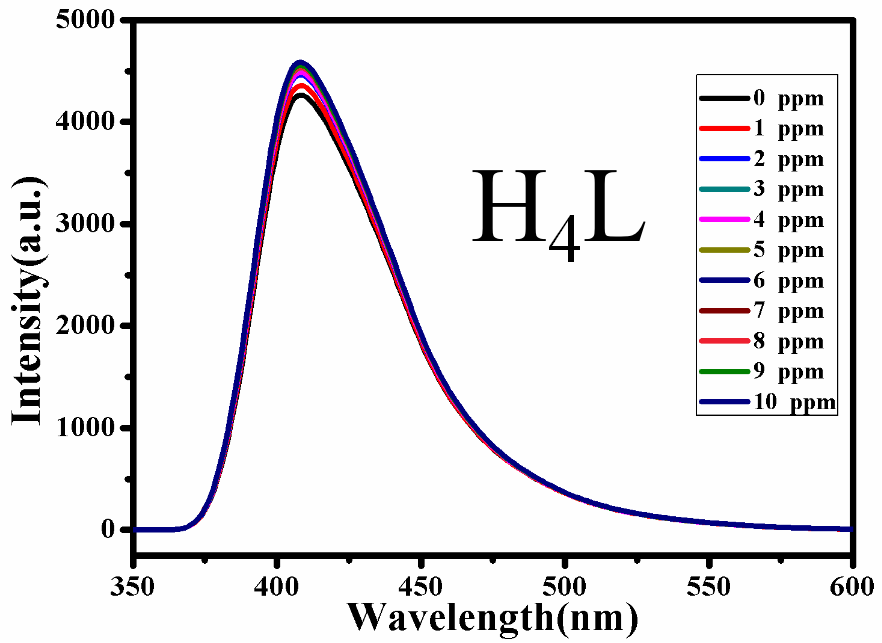


**Figure S18**. Effect on the emission spectra of H_4_**L** upon incremental addition of a 1,4-DNB solution (1mM) in DMSO.


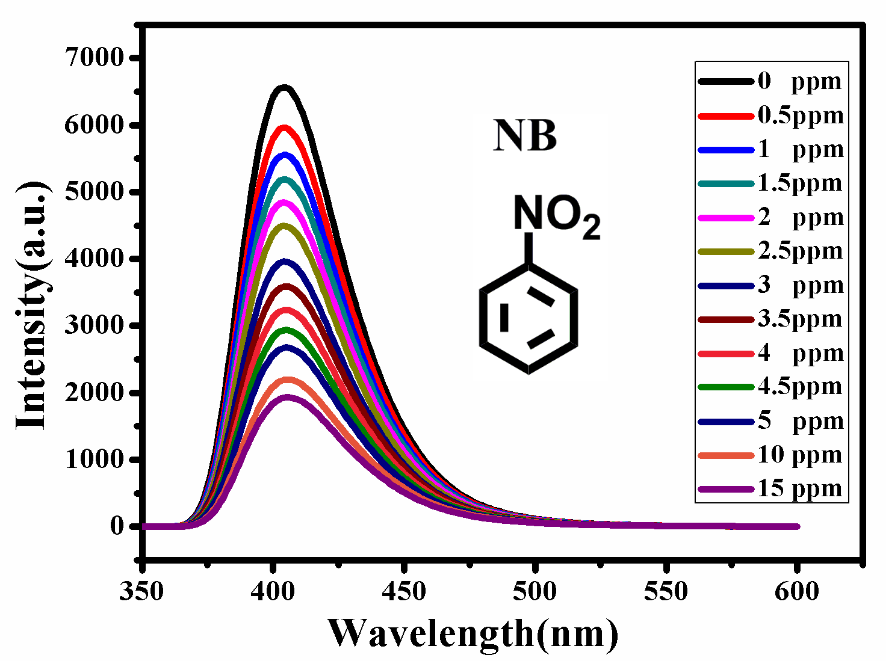


**Figure S19**. Effect on the emission spectra of **UPC-21** upon incremental addition of a DNB solution (1mM) in H_2_O.


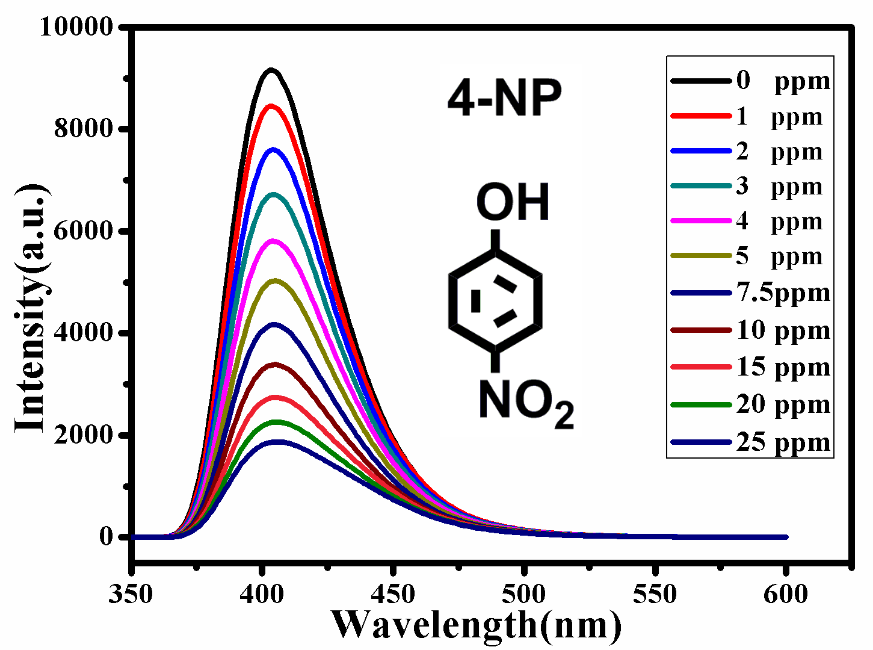


**Figure S20**. Effect on the emission spectra of **UPC-21** upon incremental addition of a 4-NP solution (1mM) in H_2_O.


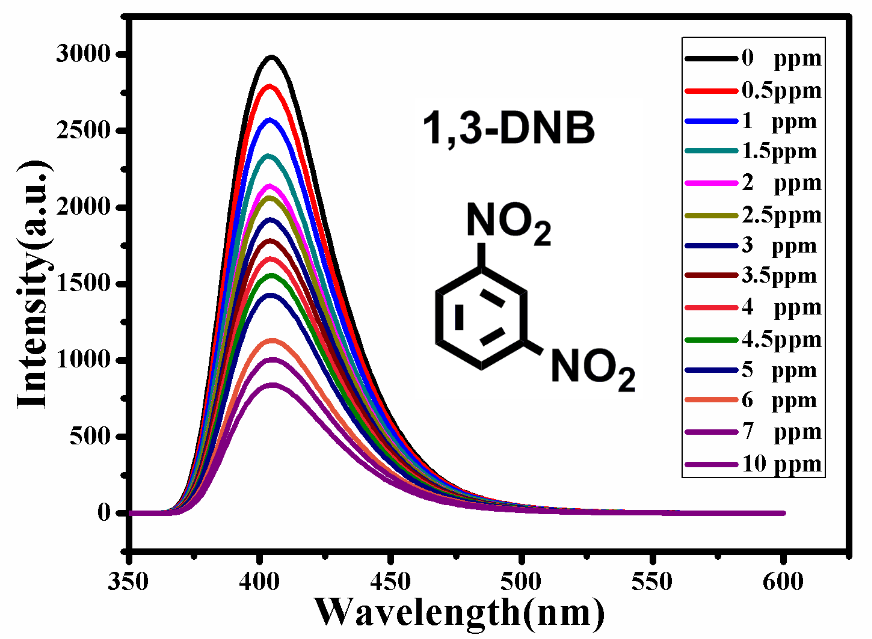


**Figure S21**. Effect on the emission spectra of **UPC-21** upon incremental addition of a 1,3-DNB solution (1mM) in H_2_O.


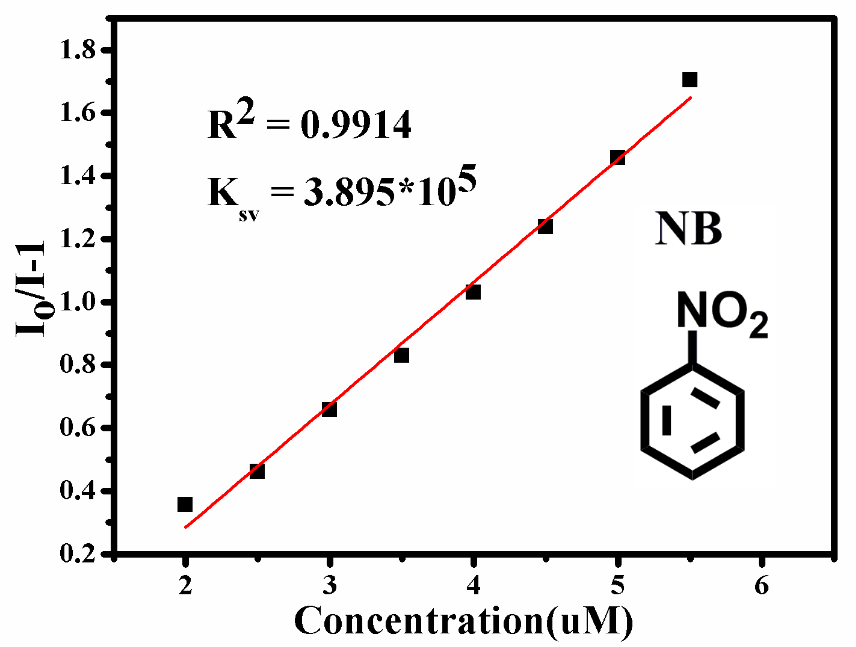


**Figure S22**. The Stern–Volmer plots for **UPC-21** with NB in Water.


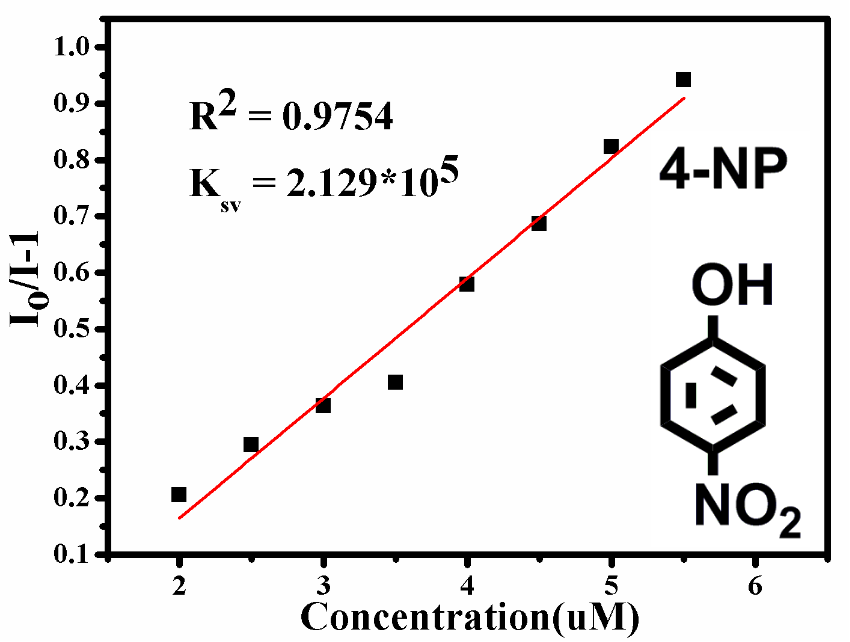


**Figure S23**. The Stern–Volmer plots for **UPC-21** with 4-NP in water.


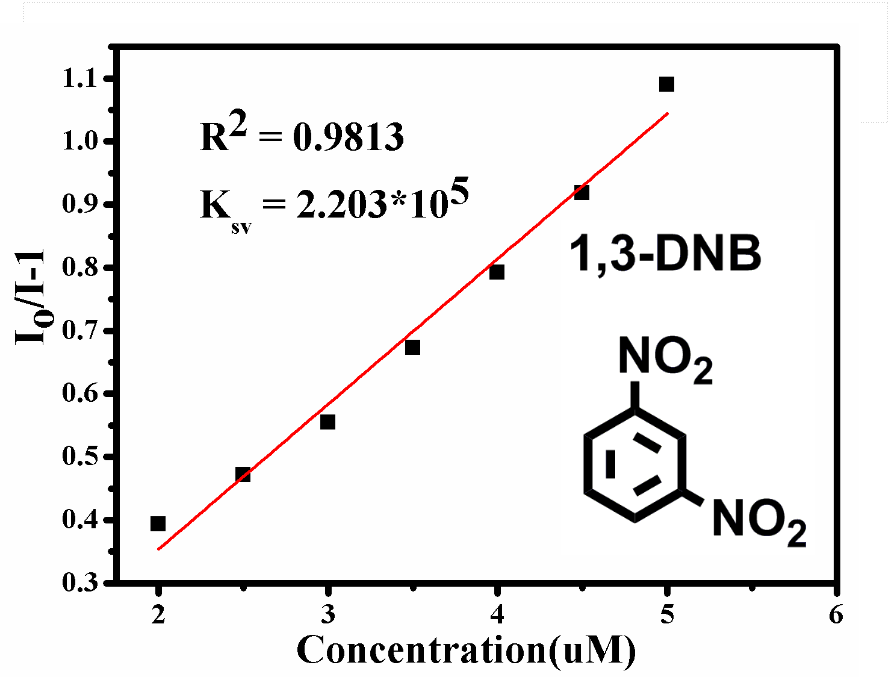


**Figure S24**. The Stern–Volmer plots for **UPC-21** with 1,3-DNB in water.

Table S4. Comparison of present result with previously reported TNP sensors.

| No. | *K*_sv_/M^-1^ | Detection limit | Reference |
| --- | --- | --- | --- |
| 1 | 3.2 × 10^6^ | 0.9ppb | S4 |
| 2 | 3.57× 10^5^ | 1ppb | S5 |
| 3 | 2.5 × 10^5^ | 0.4 ppm | S6 |
| 4 | 1.322 × 10^5^ |  | S7 |
| 5 | 1 × 10^5^ |  | S8 |
| 6 | 9.9 × 10^4^ | 2-200 ppb | S9 |
| 7 | 7.8 × 10^4^ |  | S10 |
| 8 | 6.9 × 10^4^ | 1ppm | S11 |
| 9 | 5.1 × 10^4^ | 10 ppb | S12 |
| 10 | 38134 |  | S13 |
| 11 | 3.5× 10^4^ |  | S14 |
| 12 | 3.04× 10^4^ | 23ppb | S15 |
| 13 | 2.9 × 10^4^ | 6.6ppm | S16 |
| 14 | 1.839 × 10^4^ |  | S17 |
| 15 | 1.55 × 10^4^ | 0.35ppm | S18 |
| 16 | 1.1 × 10^4^ |  | S19 |
| 17 | 9.09 × 10^3^ | 1 ppm | S20 |
| 19 | 5.7 × 10^3^ | 0.32ppm | S21 |
| 20 |  | 4.8 ppb | S22 |
| 21 |  | 2.59ppm | S23 |
| **22** | **5.209 × 10^5^** | **0.202ppm** | **UPC-21** |

**Supporting References:**

- - - 1. Yang, J., Swager, T. M. Fluorescent Porous Polymer Films as TNT Chemosensors: Electronic and Structural Effects. *J. Am. Chem. Soc*. **120**, 11864–11873 (1998).
      2. Sheldrick, G. M. SHELXL-97, *Program for X-ray Crystal Structure Refinement*; University of Gottingen: Gottingen, Germany, **1997**.
      3. Tian, D., Li, Y., Chen, R. Y., Chang, Z., Wang, G. Y., Bu, X. H. A Luminescent Metal–Organic Framework Demonstrating Ideal Detection Ability for Nitroaromatic Explosives. *J. Mater. Chem*. **2**, 1465-1470 (2014).
      4. Vij, V., Bhalla, V., Kumar, M. Attogram Detection of Picric Acid by Hexa-peri-Hexabenzocoronene-Based Chemosensors by Controlled Aggregation-Induced Emission Enhancement. *Appl. Mater. Interfaces*. **5**, 5373–5380 (2013).
      5. Kumar, R., Sandhu, S., Singh, P., Hundal, G., Hundal, M. S., Kumar, S. Tripodal Fluorescent Sensor for Encapsulation-Based Detection of Picric Acid in Water. *Asian J. Org. Chem*. **3**, 805-813 (2014).
      6. Li, D., Liu, J., Kwok, R. T., Liang, Z., Tang, B. Z., Yu, J. Supersensitive Detection of Explosives by Recyclable AIE Luminogen-Functionalized Mesoporous Materials. *Chem. Commun*. **48**, 7167-7169 (2012).
      7. Dinda, D., Gupta, A., Shaw, B. K., Sadhu, S., Saha, S. K. Highly Sensitive and Selective Detection of 2,4,6-Trinitrophenol Using Covalent-Organic Polymer Luminescent Probes. *Appl. Mater. Interfaces*. **6**, 10722–10728 (2014).
      8. Wang, M. *et al*. Trigonal Cages from Preorganized Metalloligands Incorporating Octahedral Metal Centers and Fluorescent Detection of Nitroaromatics. *Inorg. Chem*. **50**, 1506-1512 (2011).
      9. Venkatramaiah, N., Kumar, S., Patil, S. Fluoranthene Based Fluorescent Chemosensors for Detection of Explosive Nitroaromatics. *Chem. Commun*. **48**, 5007-5009 (2012).
      10. Dalapati, S., Jin, S. B., Gao, J., Xu, Y. H., Nagai, A., Jiang, D. L. An Azine-Linked Covalent Organic Framework. *J. Am. Chem. Soc.* **135**, 17310-17313 (2013).
      11. Bhalla, V., Gupta, A., Kumar, M. Fluorescent Nanoaggregates of Pentacenequinone Derivative for Selective Sensing of Picric acid in Aqueous Media. *Org. Letters*, **14**, 3112-3115 (2012).
      12. Du, H. Y., He, G., Liu, T. H., Ding, L. P., Fang, Y. J. Preparation of Pyrene-Functionalized fluorescent film with a Benzene Ring in Spacer and Sensitive Detection to Picric Acid in Aqueous Phase. *Photoche. Photobio*. **217**, 356-362 (2011).
      13. Wu, P. J., Kuo, S. Y., Huang, Y. C., Chen, C. P., Chan, Y. H. Polydiacetylene-Enclosed Near-Infrared Fluorescent Semiconducting Polymer Dots for Bioimaging and Sensing. *Anal. Chem*. **86**, 4831-4839 (2014).
      14. Nagarkar, S. S., Joarder, B., Chaudhari, A. K., Mukherjee, S., Ghosh, S. K. Highly Selective Detection of Nitro Explosives by a Luminescent Metal-Organic Framework. *Angew. Chem., Int. Ed*. **52**, 2881-2885 (2013).
      15. Ding, L. P., Liu, Y., Cao, Y., Wang, L. L., Xin, Y. H., Fang, Y. A Single Fluorescent Self-Assembled Monolayer Film Sensor with Discriminatory Power. *J. Mater. Chem*. **22**, 11574–11582 (2012).
      16. Khatua, S., Goswami, S., Biswas, S., Tomar, K., Jena, H. S., Konar, S. Stable Multiresponsive Luminescent MOF for Colorimetric Detection of Small Molecules in Selective and Reversible Manner. *Chem. Mater.* **27**, 5349-5360 (2015).
      17. Qian, J. J., Qiu, L. G., Wang, Y. M., Yuan, Y. P., Xie, A. J., Shen, Y. H. Fabrication of Magnetically Separable Fluorescent Terbium-Based MOF Nanospheres for Highly Selective Trace-level Detection of TNT. *Dalton Trans*. **43**, 3978−3983 (2014).
      18. Bhalla, V., Gupta, A., Kumar, M., Rao, D. S., Prasad, S. K. Self-Assembled Pentacenequinone Derivative for Trace Detection of Picric Acid. *ACS* *Appl. Mater. Interfaces.* **5**, 672-679 (2013).
      19. Sohn, H., Sailor, M. J., Magde, D., Trogler, W. C. Detection of Nitroaromatic Explosives Based on Photoluminescent Polymers Containing Metalloles. *J. Am. Chem. Soc.* **125**, 3821-3830 (2003).
      20. Lu, P. *et al*. Aggregation-Induced Emission in a Hyperbranched Poly(silylenevinylene) and Superamplification in Its Emission Quenching by Explosives. *Macromol. Rapid Commun*. **31**, 834–839 (2010).
      21. Liu, T. H., Ding, L. P., He, G., Yang,Y., Wang, W. L., Fang, Y. Photochemical Stabilization of Terthiophene and Its Utilization as a New Sensing Element in the Fabrication of Monolayer-Chemistry-Based Fluorescent Sensing Films. *ACS* *Appl. Mater. Interfaces.* **3**, 1245-1253 (2011).
      22. He, G., Peng, H. N., Liu, T. H., Yang, M. N., Zhang, Y., Fang, Y. A Novel Picric Acid Film Sensor via Combination of the Surface Enrichment Effect of Chitosan Films and the Aggregation-Induced Emission Effect of Siloles. *J. Mater. Chem*. 19, 7347-7353 (2009).
      23. Ye, J. W. *et al*. A Fluorescent Zinc–Pamoate Coordination Polymer for Highly Selectivesensing of 2,4,6-Trinitrophenol and Cu^2+^ion. *Sens. Act.* **210**, 566-573 (2015).
